# A guide to selecting high-performing antibodies for Netrin-1 (UniProt ID: O95631) for use in western blot and immunoprecipitation

**DOI:** 10.12688/f1000research.184099.1

**Published:** 2026-07-10

**Authors:** Riham Ayoubi, Sara González Bolívar, Richard A Kahn, Vincent Francis, Peter S. McPherson, Carl Laflamme, NeuroSGC/YCharOS/EDDU collaborative group and ABIF consortium

**Affiliations:** 1 Department of Neurology and Neurosurgery, Structural Genomics Consortium, The Montreal Neurological Institute, McGill University, Montreal, Canada

**Keywords:** O95631, NTN1, Netrin-1, antibody characterization, antibody validation, western blot, immunoprecipitation, immunofluorescence

## Abstract

Netrin-1 is a secreted protein that regulates cell migration and survival, controlling axonal guidance during development, morphogenesis, apoptosis, angiogenesis, inflammation and cancer progression. Here we have characterized fourteen Netrin-1 commercial antibodies for western blot and immunoprecipitation using a standardized experimental protocol based on comparing read-outs in knockout cell lines and isogenic parental controls. These studies are part of a larger, collaborative initiative seeking to address antibody reproducibility issues by characterizing commercially available antibodies for human proteins and publishing the results openly as a resource for the scientific community. While the use of antibodies and protocols vary between laboratories, we encourage readers to use this report as a guide to select the most appropriate antibodies for their specific needs.

## Introduction

Netrin-1 encoded by the *NTN1* gene belongs to the Netrins family of laminin-like proteins (the laminin superfamily) typically involved in cell and axon migration during embryogenesis. ^1^ Netrin-1 is a secreted protein that consists of 604 amino acids and contains an N-terminal laminin-like domain, three epidermal growth factor-like repeats responsible for binding to the receptors, and a C-terminal Netrin-like module which contributes to its secretion. ^2^ ^,^ ^3^ During embryonic development, Netrin-1 guides axonal growth, neuron migration and glial differentiation. In non-neural organs, Netrin-1 plays an important role in tissue morphogenesis of mammary glands, lungs and pancreas. ^4^ It also acts as an apoptotic inhibitor during angiogenesis, impacting survival of the cells. ^5^ Netrin-1 also modulates acute and chronic inflammation mechanisms to protect the cells ^6^ and is dysregulated in multiple cancers, indicating its involvement in tumorigenesis regulation. ^3^ Lastly, Parkinson’s disease patients exhibit reduced levels of Netrin-1 as compared to control, and the loss of this protein may be contributing to the disease progression. ^7^

This research is part of a broader collaborative initiative in which academics, funders and commercial antibody manufacturers are working together to address antibody reproducibility issues by characterizing commercial antibodies for human proteins using standardized protocols, and openly sharing the data. ^8^ It consists of identifying human cell lines with adequate target protein expression and the development/contribution of equivalent knockout (KO) cell lines, followed by antibody characterization procedures using most commercially available antibodies against the corresponding protein. ^8^ Here we characterized fourteen commercial Netrin-1 antibodies, selected and donated by participant antibody manufacturers, for use in western blot and immunoprecipitation, enabling biochemical and cellular assessment of Netrin-1 properties and function.

The authors do not engage in result analysis or offer explicit antibody recommendations. Our primary aim is to deliver top-tier data to the scientific community, grounded in Open Science principles. This empowers experts to interpret the characterization data independently, enabling them to make informed choices regarding the most suitable antibodies for their specific experimental needs. Guidelines on how to interpret antibody characterization data found in this study are featured on the YCharOS gateway ^9^ and in Table 3 of this data note. ^8^ Guidelines on how to interpret antibody characterization data found in this study are featured on the YCharOS gateway ^9^ and in Table 3 of this data note. ^8^

## Results and discussion

Our standard protocol involves comparing readouts from wild type (WT) and KO cell lines. ^10^ ^,^ ^11^ A commercially available *NTN1* KO cell line in MCF7 was obtained from Abcam and was used for this study ( Table 1). DepMap (Cancer Dependency Map Portal, RRID: SCR_017655) is a transcriptomics database which helps identify cell lines that express the target at levels greater than 2.5 log _2_ (transcripts per million “TPM” + 1), which we have found to be a suitable cut-off. ^12^ Since MCF7 expresses the *NTN1* transcript at 1.6 log _2_ TPM + 1, U-2 OS was selected as a second cell line to KO the corresponding *NTN1* gene using CRISPR/Cas9 ( Table 1).

**Table 1.** Summary of the cell lines used.

Institution	Catalog number	RRID (Cellosaurus)	Cell line	Genotype
Abcam	ab271144	CVCL_0031	MCF7	WT
Abcam	ab323856	CVCL_E8G1	MCF7	*NTN1* KO
ATCC	HTB-96	CVCL_0042	U-2 OS	WT
Richard Kahn (line available upon request)	-	CVCL_E8G2	U-2 OS	*NTN1* KO
ATCC	HTB-14	CVCL_0022	U-87 MG	WT
ATCC	CCL-2	CVCL_0030	HeLa	WT
ATCC	CCL-121	CVCL_0317	HT-1080	WT
ATCC	CRL-2142	CVCL_1702	SK-N-FI	WT
ATCC	CCL-185	CVCL_0023	A549	WT
ATCC	CRL-1555	CVCL_0037	A-431	WT
ATCC	CRL-3216	CVCL_0063	293 T	WT
ATCC	HTB-161	CVCL_0465	OVCAR3	WT

**Table 2.** Summary of the Netrin-1 antibodies tested.

Company	Catalog number	Lot number	RRID (Antibody Registry)	Clonality	Clone ID	Host	Concentration (μg/μL)	Vendors recommended applications
Abcam	ab126729 **	1085976–3	AB_11131145	recombinant mono	EPR5428	rabbit	1.65	Wb
Bio-Techne (Novus Biologicals)	NBP3–21882 **	231062	AB_3076156	recombinant mono	SR2201	rabbit	0.00	Wb, IF
Bio-Techne (Novus Biologicals)	NBP3–32643 **	H681262021	NA	recombinant mono	JE38–87	rabbit	1.00	Wb
Bio-Techne (R&D Systems)	AF6419	CFXN0322071	AB_3075874	polyclonal	-	sheep	0.20	Wb
Bio-Techne (R&D Systems)	MAB1109 *	GCT0222061	AB_2154710	monoclonal	158936	rat	0.50	Wb
GeneTex	GTX133213	42732	AB_2886871	polyclonal	-	rabbit	0.53	Wb
Proteintech	20235–1-AP	00094636	AB_2918065	polyclonal	-	rabbit	0.50	Wb
Thermo Fisher Scientific	PA5–98017	YJ4089029	AB_2812631	polyclonal	-	rabbit	4.88	Wb
Institute for Protein Innovation	IPIab00000283 **	3	AB_3698382	recombinant mono	IPI-Netrin.33	rabbit	0.50	NA
Institute for Protein Innovation	IPIab00000284 **	3	AB_3698383	recombinant mono	IPI-Netrin.43	rabbit	0.50	NA
Institute for Protein Innovation	IPIab00000285 **	3	AB_3751928	recombinant mono	IPI-Netrin.45	rabbit	0.50	NA
Institute for Protein Innovation	IPIab00000286 **	3	AB_3698384	recombinant mono	IPI-Netrin.55	rabbit	0.50	NA
Institute for Protein Innovation	IPIab00000287 **	3	NA	recombinant mono	IPI-Netrin.58	rabbit	0.50	NA
Institute for Protein Innovation	IPIab00000288 **	3	NA	recombinant mono	IPI-Netrin.65	rabbit	0.50	NA

Wb=western blot; IF= immunofluorescence,

** = recombinant antibody,

* = monoclonal antibody, NA=not available.

**Figure 1.**
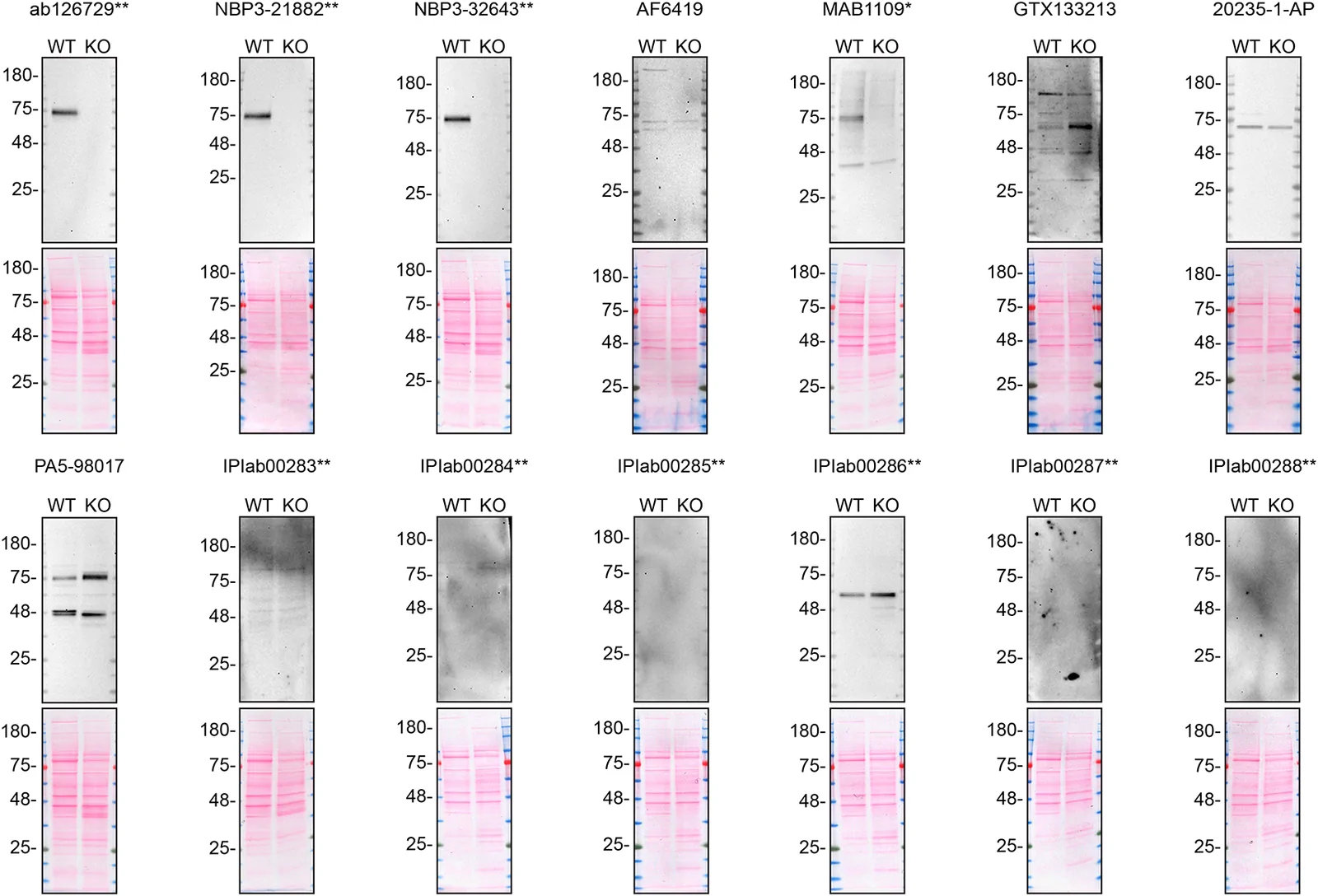
Netrin-1 antibody screening by western blot on MCF7 culture medium. Culture media from MCF7 WT and *NTN1* KO were collected, and 10 μg of protein were processed for western blot with the indicated Netrin-1 antibodies. The Ponceau stained transfers of each blot are presented to show equal loading of WT and KO samples and protein transfer efficiency from the acrylamide gels to the nitrocellulose membrane. Antibody dilutions were chosen according to the recommendations of the antibody supplier. Antibody dilutions used: ab126729** at 1/2000, NBP3–21882** at 1/1000, NBP3–32643** at 1/1000, AF6419 at 1/30, MAB1109* at 1/500, GTX133213 at 1/500, 20235–1-AP at 1/3000, PA5–98017 at 1/3000, IPIab00000283** at 1/100 (5 μg/mL), IPIab00000284** at 1/100, IPIab00000285** at 1/100, IPIab00000286** at 1/100, IPIab00000287** at 1/100, and IPIab00000288** at 1/100. Predicted band size: 67.7 kDa. ** = recombinant antibody, * = monoclonal antibody.

**Figure 2.**
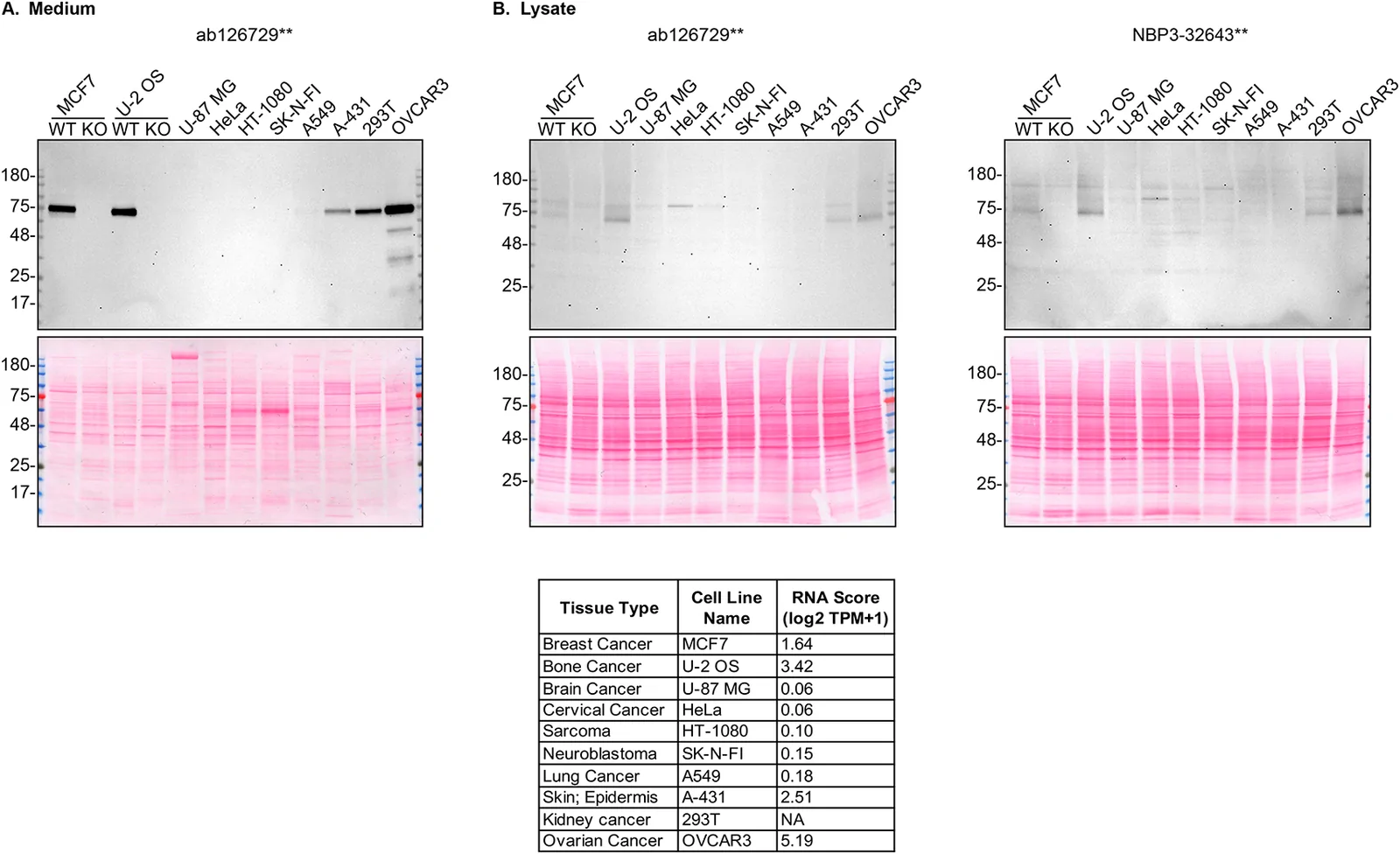
Netrin-1 western blot on culture media and lysates from various human cell lines. **A)** Culture media were collected from WT and *NTN1* KO MCF7, WT and *NTN1* KO U-2 OS, WT U-87 MG, HeLa, HT-1080, SK-N-FI, A549, A-431, 293 T and OVCAR3, and 10 μg of protein were processed for western blot with anti-Netrin-1 ab126729** at 1/2000. **B)** Lysates were prepared from WT and *NTN1* KO MCF7, WT U-2 OS, U-87 MG, HeLa, HT-1080, SK-N-FI, A549, A-431, 293 T and OVCAR3, and 30 μg of protein were processed for western blot with anti-Netrin-1 ab126729** at 1/2000 and NBP3–21882** at 1/1000. ** = recombinant antibody.

To detect secreted Netrin-1, all fourteen antibodies were first screened by western blot with the commercially accessible MCF7 WT and *NTN1* KO proteins from culture medium ran on SDS-PAGE, transferred onto nitrocellulose membranes, and then probed with the fourteen Netrin-1 antibodies in parallel ( Figure 1). Next, the differrential expression of Netrin-1 was evaluated by western blot in culture media from various cell types ( Figure 2A) at different RNA levels ( Figure 2). U-2 OS WT and *NTN1* KO were included in this cell panel and showed similar levels of secreted Netrin-1 in MCF7 and U-2 OS. Netrin-1 protein levels were also assessed in lysates from those same cell lines to explore the availability of the protein intracellularily prior to immunofluorescence. Intracellular Netrin-1 was detected at much lower levels than the secreted protein and required a higher amount of protein to be loaded onto the gel as well as significantly higher exposure times to attain detectable signal using two different antibodies in western blot ( Figure 2B). The immunofluorescence assay was thus dismissed in this data note.

**Figure 3.**
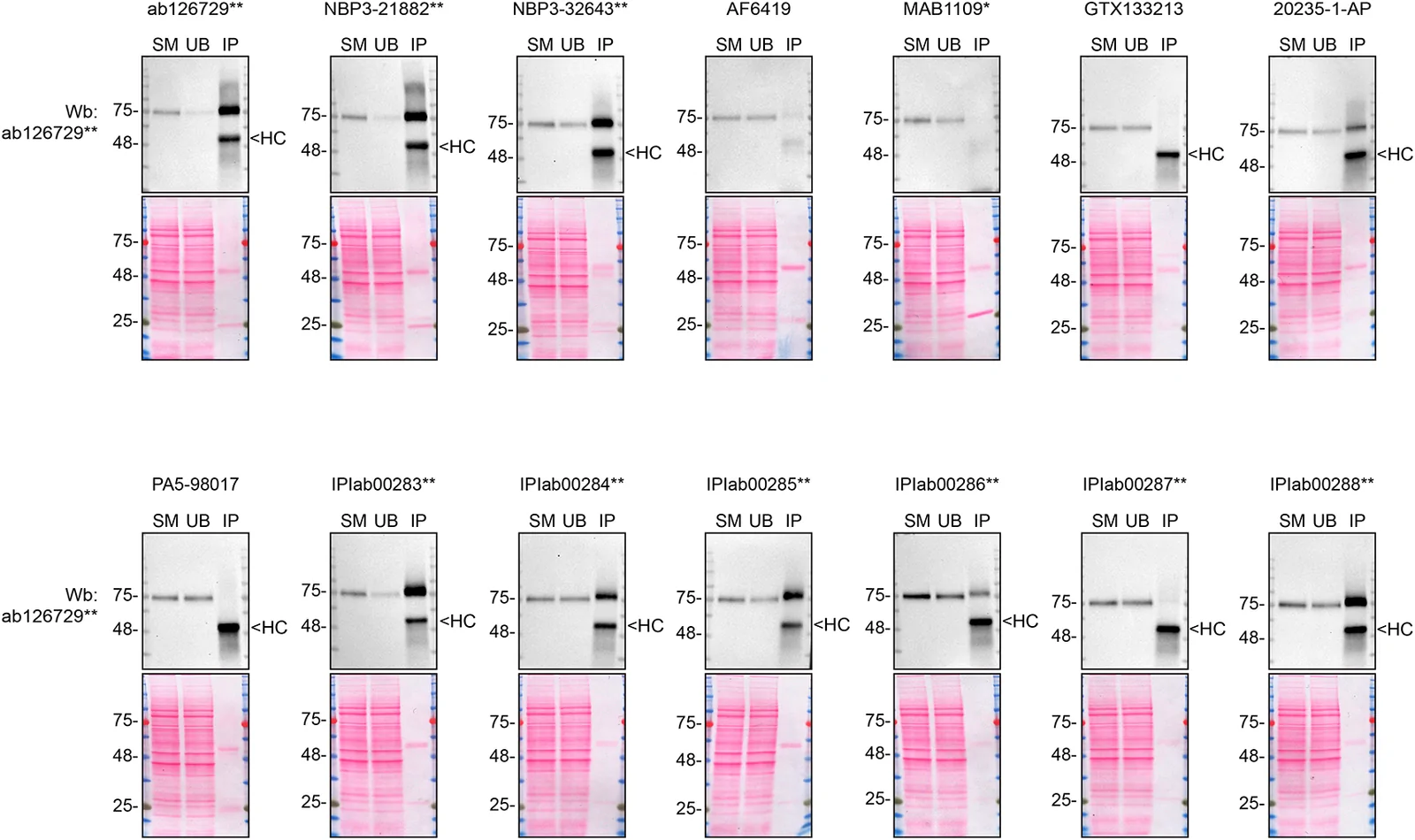
Netrin-1 antibody screening by immunoprecipitation on MCF7 culture medium. Culture medium was collected from MCF7, and immunoprecipitation was performed for 1 h using 0.15 mg of protein and 2.0 μg of the indicated Netrin-1 antibodies pre-coupled to Dynabeads protein A or protein G. Samples were washed and processed for western blot with the anti-Netrin-1 ab126729** diluted at 1/2000. The Ponceau stained transfers of each blot are shown. SM = 10% starting material; UB = 10% unbound fraction; IP = immunoprecipitate; HC = antibody heavy chain. ** = recombinant antibody, * = monoclonal antibody.

We then assessed the capability of all fourteen antibodies to capture Netrin-1 from MCF7 culture medium using the immunoprecipitation technique, followed by western blot analysis. For the immunoblot step, a specific Netrin-1 antibody identified previously (refer to Figure 1) was selected. Equal amounts of the starting material (SM) and the unbound fractions (UB), as well as the whole immunoprecipitate (IP) eluates were separated by SDS-PAGE ( Figure 3).

In conclusion, we have screened fourteen Netrin-1 commercial antibodies by western blot and immunoprecipitation by comparing the signal produced by the antibodies in human MCF7 WT and *NTN1* KO cells. To assist users in interpreting antibody performanyce, Table 3 outlines various scenarios in which antibodies may perform in these applications. ^12^ Several high-quality and renewable antibodies that successfully detect Netrin-1 were identified in both applications. Researchers who wish to study Netrin-1 in a different species are encouraged to select high-quality antibodies, based on the results of this study, and investigate the predicted species reactivity of the manufacturer before extending their research.

**Table 3.** Illustrations to assess antibody performance in all western blot and immunoprecipitation.

Western blot	Immunoprecipitation
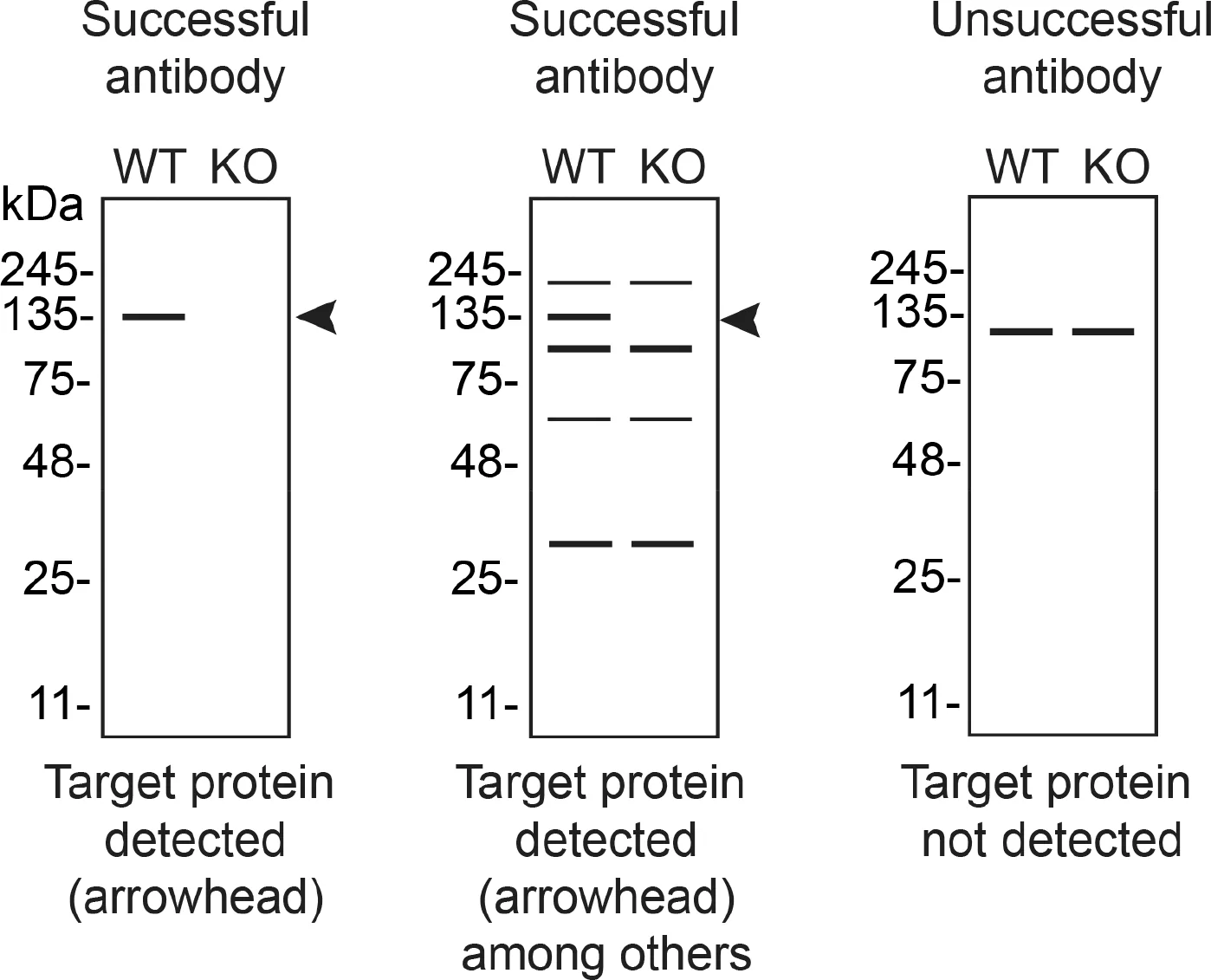	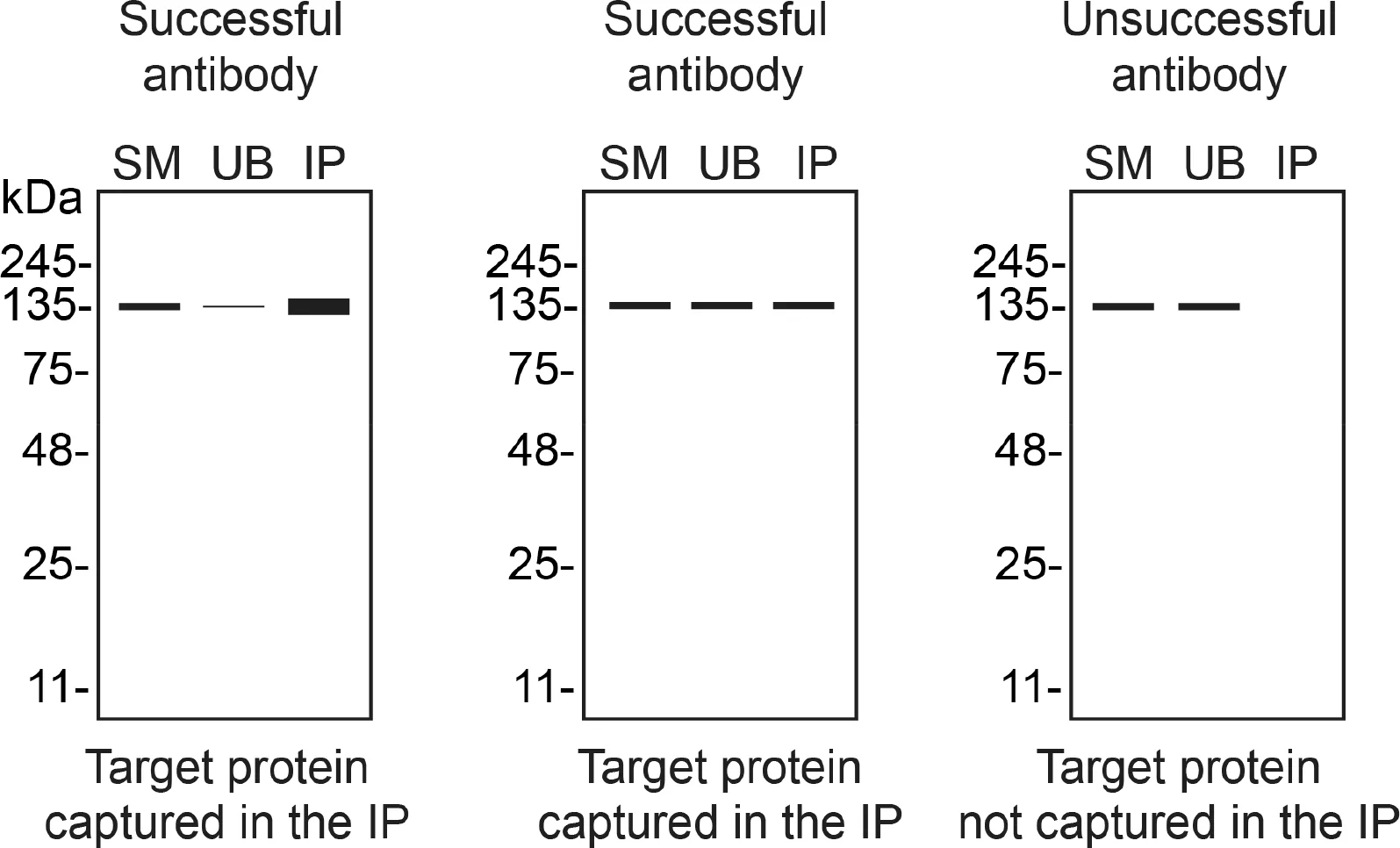

This table has been reproduced with permission from Ayoubi et al., Elife, 2023. ^12^

## Limitations

Inherent limitations are associated with the antibody characterization platform used in this study. Firstly, the YCharOS project focuses on renewable (recombinant and monoclonal) antibodies and does not test all commercially available Netrin-1 antibodies. YCharOS partners provide approximately 80% of all renewable antibodies, but some top-cited polyclonal antibodies may not be available through these partners.

Secondly, the YCharOS effort employs a non-biased approach that is agnostic to the protein for which antibodies have been characterized. The aim is to provide objective data on antibody performance without preconceived notions about how antibodies should perform or the molecular weight that should be observed in western blot. As the authors are not experts in Netrin-1, only a brief overview of the protein’s function and its relevance in disease is provided. Netrin-1 experts are invited to analyze and interpret observed banding patterns in western blots and subcellular localization in immunofluorescence.

Thirdly, YCharOS experiments are not performed in replicates primarily due to the use of multiple antibodies targeting various epitopes. Once a specific antibody is identified, it validates the protein expression of the intended target in the selected cell line, confirms the lack of protein expression in the KO cell line and supports conclusions regarding the specificity of the other antibodies. All experiments are performed using master mixes, and meticulous attention is paid to sample preparation and experimental execution. In IF, the use of two different concentrations serves to evaluate antibody specificity and can aid in assessing assay reliability. In instances where antibodies yield no signal, a repeat experiment is conducted following titration. Additionally, our independent data is performed subsequently to the antibody manufacturers internal validation process, therefore making our characterization process a repeat.

Lastly, as comprehensive and standardized procedures are respected, any conclusions remain confined to the experimental conditions and cell line used for this study. The use of a single cell type for evaluating antibody performance poses as a limitation, as factors such as target protein abundance significantly impact results. Additionally, the use of cancer cell lines containing gene mutations poses a potential challenge, as these mutations may be within the epitope coding sequence or other regions of the gene responsible for the intended target. Such alterations can impact the binding affinity of antibodies. This represents an inherent limitation of any approach that employs cancer cell lines.

## Method

The standardized protocols used to carry out this KO cell line-based antibody characterization platform was established and approved by a collaborative group of academics, industry researchers and antibody manufacturers. The detailed materials and step-by-step protocols used to characterize antibodies in western blot, immunoprecipitation and immunofluorescence are openly available on Protocols.io ( protocols.io/view/a-consensus-platform-for-antibody-characterization ). ^8^ Brief descriptions of the experimental setup used to carry out this study can be found below.

### Cell lines and antibodies

Cell lines used and primary antibodies tested in this study are listed in Tables 1 and 2, respectively. To ensure that the cell lines and antibodies are cited properly and can be easily identified, we have included their corresponding Research Resource Identifiers, or RRID. ^13^ ^,^ ^14^

Peroxidase-conjugated goat anti-rabbit and anti-rat antibodies are from Thermo Fisher Scientific, cat. Number 65–6120 and 31470, respectively. Peroxidase-conjugated donkey anti-sheep is from Thermo Fisher Scientific, cat. Number A16041. Peroxidase-conjugated Protein A for IP detection is from Cell Signaling Technology, cat. Number 12291.

### Antibody screening by western blot

MCF7 WT and *NTN1* KO cells were washed 3x with PBS 1x and starved for ~36 hrs. Culture media were collected and centrifuged for 10 min at 500 x *g* to eliminate cells and larger contaminants, then for 10 min at 4500 x *g* to eliminate smaller contaminants. Culture media were then concentrated by centrifuging at 4000 x *g* for 30 min using the Amicon Ultra-15 Centrifugal Filter Units with a membrane NMWL of 10 kDa (MilliporeSigma cat. Number UFC901024). Culture media were finally supplemented with 1x protease inhibitor cocktail mix (MilliporeSigma, cat. Number P8340). BLUelf prestained protein ladder (GeneDireX, cat. Number PM008–0500) was used.

Western blots were performed with precast midi 4–20% Tris-Glycine polyacrylamide gels (Thermo Fisher Scientific, cat. Number WXP42012BOX) ran with Tris/Glycine/SDS buffer (Bio-Rad, cat. Number 1610772), loaded in Laemmli loading sample buffer (Thermo Fisher Scientific, cat. Number AAJ61337AD) and transferred on nitrocellulose membranes. Proteins on the blots were visualized with Ponceau S staining (Thermo Fisher Scientific, cat. Number BP103–10) which is scanned to show together with individual western blot. Blots were blocked with 5% milk for 1 hr, and antibodies were incubated O/N at 4 °C with 5% milk in TBS with 0,1% Tween 20 (TBST) (Cell Signalling Technology, cat. Number 9997). Following three washes with TBST, the peroxidase conjugated secondary antibody was incubated at a dilution of ~0.2 μg/mL in TBST with 5% milk for 1 hr at room temperature followed by three washes with TBST. Membranes were incubated with Pierce ECL (Thermo Fisher Scientific, cat. Number 32106) prior to detection with the iBright™ CL1500 Imaging System (Thermo Fisher Scientific, cat. Number A44240).

For lysate preparation, cells were collected in RIPA buffer (25 mM Tris-HCl pH 7.6, 150 mM NaCl, 1% NP-40, 1% sodium deoxycholate, 0.1% SDS) (Thermo Fisher Scientific, cat. Number 89901) supplemented with 1x protease inhibitor cocktail mix. Lysates were sonicated briefly and incubated 30 min on ice. Lysates were spun at ~110,000 *x g* for 15 min at 4 °C and equal protein aliquots of the supernatants were analyzed by SDS-PAGE and western blot.

### Antibody screening by immunoprecipitation

Antibody-bead conjugates were prepared by adding 2 μg of antibody to 500 μl of Pierce IP Lysis Buffer (25 mM Tris-HCl pH 7.4, 150 mM NaCl, 1 mM EDTA, 1% NP-40 and 5% glycerol) from Thermo Fisher Scientific, cat. Number 87788 in a microcentrifuge tube, together with 30 μl of Dynabeads protein A- (for rabbit antibodies) or protein G- (for rat and sheep antibodies) (Thermo Fisher Scientific, cat. Number 10002D and 10004D, respectively). NBP3–21882** was at an unknown concentration and therefore 2 μl were tested in the IP. Tubes were rocked for ~1 h at 4 °C followed by two washes to remove unbound antibodies.

Culture media from MCF7 WT were collected as described in the western section above. 0.15 mL aliquots at 1 mg/mL of culture medium were incubated with an antibody-bead conjugate for ~1 h at 4 °C. The unbound fractions were collected, and beads were subsequently washed three times with 1.0 mL of IP buffer and processed for SDS-PAGE and western blot on precast midi 4–20% Tris-Glycine polyacrylamide gels. Protein A:HRP was used as a secondary detection system at a concentration of 0.5 μg/mL.

## Data Availability

Zenodo: Dataset for the Netrin-1 antibody screening study. https://doi.org/10.5281/zenodo.20533820. Data are available under the terms of the Creative Commons Attribution 4.0 International license (CC-BY 4.0).
