# Supplementary material for: A guide to selecting high-performing antibodies for Netrin-1 (UniProt ID: O95631) for use in western blot and immunoprecipitation
Source: F1000Res. 2026 Jul 10;15:1129. [Version 1] doi: 10.12688/f1000research.184099.1 (PMC13583349; doi:10.12688/f1000research.184099.1)
Supplement: Peer review history

# Reviewer report: Reviewer response for version 1

**DOI:** 10.5256/f1000research.203211.r512301

**Published:** 2026-09-15

**Authors:** Jieya Shao

**Affiliations:** 1 Washington University in St Louis, St. Louis, USA

In this article the authors systematically compared the performance and specificity of fourteen commercial anti-Netrin-1 antibodies using two standard experimental approaches (Western blot and immunoprecipitation) to detect Netrin-1 in secreted cell medium and intracellular lysates. This study is part of a large collaborative initiative to characterize commercial antibodies for different human proteins and offer unbiased guidance to researchers who can select the best antibodies for their specific experimental needs. As a Data Note, this paper successfully achieved its goal with carefully collected and clearly presented high-quality data, and the results are useful for the research community.

Are sufficient details of methods and materials provided to allow replication by others?

Yes

Is the rationale for creating the dataset(s) clearly described?

Yes

Are the datasets clearly presented in a useable and accessible format?

Yes

Are the protocols appropriate and is the work technically sound?

Yes

Reviewer Expertise:

cancer biology, genome integrity maintenance

I confirm that I have read this submission and believe that I have an appropriate level of expertise to confirm that it is of an acceptable scientific standard.

# Reviewer report: Reviewer response for version 1

**DOI:** 10.5256/f1000research.203211.r512302

**Published:** 2026-09-11

**Authors:** Karen Bowman

**Affiliations:** 1 University of Leicester, Leicester, England, UK

The study involved characterisation of fourteen commercial antibodies, for the Netrin-1 protein, encoded by the *NTN1* gene, for use in western blot and immunoprecipitation. The study formed part of a wider YCharOS collaboration to characterise commercial antibodies, which are renewable (recombinant and monoclonal), for human proteins using standardised protocols with the intention of improving antibody reproducibility issues. As a Data Note, it allows the direct comparison of the performance of commercially available antibodies to the Netrin-1 protein to aid scientists in choosing the most appropriate antibody for their studies. For example, in understanding how disease-related mutations affect the protein’s biochemical and cellular characteristics. This comparison would be of particular use in the fields of neuroscience, developmental biology, cancer, cell migration and tissue repair.

 A committee of industry and academic representatives have endorsed the platform used, and the protocols were consistent with those in general use. The platform consisted of identification of human cell lines suitable for antibody characterisation studies i.e., with adequate levels of the Netrin-1 protein to generate a measurable signal. Using the DepMap (Cancer Dependency Map Portal, RRID: SCR_017655) transcriptomics database, usually the team would select a commercially available cell line expressing the NTN1 transcript at a level above the average range for cancer cells analysed. However, none were found, therefore, two cell lines with modest levels of the Netrin-1 protein (below the usual threshold level), MCF7 and U-2 OS were chosen for study. A commercially available MCF-7 cell line (from Abcam) with knockdown of the target gene was used to provide an appropriate negative control (MCF7 *NTN1 NO*) and the *NTN1* KO of the U-2 OS cell line was achieved by CRISPR/Cas9 knock out of the *NTN1* gene in U-2 OS cells.

 The final step was a series of antibody characterisation procedures, limited to the most common research uses of antibodies. The interpretation of the results was left to the reader, with the study providing unbiased guidance. However, it gave some indication of the optimum dilutions of commercial antibodies. Limitations of the study were clearly stated.

 Since Netrin-1 is a secreted protein, the specificity of the antibodies to detect the protein in culture medium was assayed by western blot in the first instance. Using two specific antibodies (from two different manufacturers) identified in the previous assay, the differential expression of Netrin-1 was studied in a panel of cell lines, with differing RNA levels, in cell lysates. Differential expression of Netrin-1 and its secretion into culture medium was also assayed in the same cell line panel using one specific antibody. The extremely low levels of intracellular Netrin-1 detected compared to that secreted into culture medium, as well as the high total protein amounts of cell lysates required for gel loading, and the significantly higher exposure times required to attain detectable signals for cell lysates by western blot suggested that immunofluorescence assays would not be informative and were therefore not done in this study.

 Just a couple of typing errors. In the Introduction, the final sentence (Guidelines on how…) needs to be removed as it is a direct repeat of the previous sentence.

 In the results and discussion section, final paragraph, second sentence, there is a spelling mistake, it should read, performance.

Are sufficient details of methods and materials provided to allow replication by others?

Yes

Is the rationale for creating the dataset(s) clearly described?

Yes

Are the datasets clearly presented in a useable and accessible format?

Yes

Are the protocols appropriate and is the work technically sound?

Yes

Reviewer Expertise:

Cell biology, DNA damage and repair, anti-cancer drug discovery and development, diagnostics.

I confirm that I have read this submission and believe that I have an appropriate level of expertise to confirm that it is of an acceptable scientific standard.
